# Direct supplementation with Urolithin A overcomes limitations of dietary exposure and gut microbiome variability in healthy adults to achieve consistent levels across the population

**DOI:** 10.1038/s41430-021-00950-1

**Published:** 2021-06-11

**Authors:** Anurag Singh, Davide D’Amico, Pénélope A. Andreux, Gillian Dunngalvin, Timo Kern, William Blanco-Bose, Johan Auwerx, Patrick Aebischer, Chris Rinsch

**Affiliations:** 1 Amazentis SA, EPFL Innovation Park, Lausanne, Switzerland; 2 Atlantia Food Clinical Trials, Cork, Ireland; 3 Clinical Microbiomics, København, Denmark; 4 Laboratory of Integrative Systems Physiology, Ecole Polytechnique Fédérale de Lausanne, Lausanne, Switzerland; 5 Ecole Polytechnique Fédérale de Lausanne, Lausanne, Switzerland

**Keywords:** Translational research, Biomarkers

## Abstract

**Background:**

Urolithin A (UA) is produced by gut microflora from foods rich in ellagitannins. UA has been shown to improve mitochondrial health preclinically and in humans. Not everyone has a microbiome capable of producing UA, making supplementation with UA an appealing strategy.

**Objective:**

This is the first detailed investigation of the prevalence of UA producers in a healthy population and the ability of direct UA supplementation to overcome both microbiome and dietary variability. Dietary intake of a glass of pomegranate juice (PJ) was used to assess UA producer status (*n* = 100 participants) and to characterize differences in gut microbiome between UA producers from non-producers.

**Methods:**

Subjects were randomized (1:1) to either PJ or a food product containing UA (500 mg). Prevalence of UA producers and non-producers were determined in the PJ group. Diet questionnaires and fecal samples were collected to compare differences between UA producers and non-producers along with plasma samples at different time points to assess levels of UA and its conjugates between the interventions.

**Results:**

Only 12% of subjects had detectable levels of UA at baseline. Following PJ intake ~40% of the subjects converted significantly the precursor compounds into UA. UA producers were distinguished by a significantly higher gut microbiome diversity and ratio of Firmicutes to Bacteroides. Direct supplementation with UA significantly increased plasma levels and provided a >6-fold exposure to UA vs. PJ (*p* < 0.0001).

**Conclusions:**

Differences in gut microbiome and diet that dictate natural exposure to UA can be overcome via direct dietary UA supplementation.

## Introduction

Urolithin A (UA) is produced endogenously by human gut bacteria exposed to dietary polyphenolic compounds that include ellagic acid (EA) and ellagitannins (ET), such as punicalagin [1]. These polyphenolic precursors are found widely in fruits (pomegranate and certain berries) and nuts (walnuts and pecans). ET are converted to EA in the upper portion of the human gastrointestinal tract, and further metabolized by gut microflora in the large intestine into compounds known as urolithins, of which UA is among the most common [2, 3]. Individuals show large differences in urolithin production capacity due to variations in the microbiome responsible for ET metabolism [4]. Urolithins, including UA, are absorbed and conjugated in the liver and are subsequently excreted in urine and feces. Several studies have shown that UA and its two main detectable metabolites, UA glucuronide and UA sulfate, are the predominant urolithin forms in circulation [5, 6].

UA has previously been shown to activate mitophagy, the recycling of defective cellular mitochondria, and to improve mitochondrial health in pre-clinical models of aging [7] and in a first-in-human randomized, double-blind, placebo-controlled trial in elderly, sedentary subjects [6]. The benefits of UA have also been observed preclinically in several health indications, including neurodegenerative disorders, IBD, and metabolic dysfunction [8–10].

A few interventional studies have investigated the presence of urolithin metabolites after intake of dietary fruits (strawberry and raspberry) and nuts (walnuts) in different study populations [11, 12]. Investigators have also examined the association between different urolithin plasma profiles and health benefits, such as lowering cardiovascular risk in overweight populations [2, 13, 14]. However, there are limited data in large and diverse age groups in healthy adult populations describing the frequency and extent of people’s natural ability to convert ET and EA into UA such as after drinking a glass of pomegranate juice (PJ) or eating the recommended quantities of nuts and berries.

This report details the results from a clinical study in American adults (aged 20–80 years) that estimates the relative prevalence of UA producers and non-producers following a standardized dietary challenge with PJ. Metagenomic profiles of the fecal samples of volunteers distinguish the gut microbiome of UA producers from non-producers. The primary objective of the study was to compare the levels of UA and its conjugates (UA glucuronide being the main metabolite) in the circulation over time during a randomized, crossover study in which participants were orally administered either 100% PJ containing a natural profile of UA precursor compounds to a nutritional product containing a calibrated dose of 500 mg of UA. The rationale of doing such a comparison was to document the variability and low levels of UA observed via natural exposure to diet rich in precursors vs. the uniform and consistent exposure obtained via direct supplementation with UA.

## Methods

### Study design and participant demographics

**Fig. 1:**
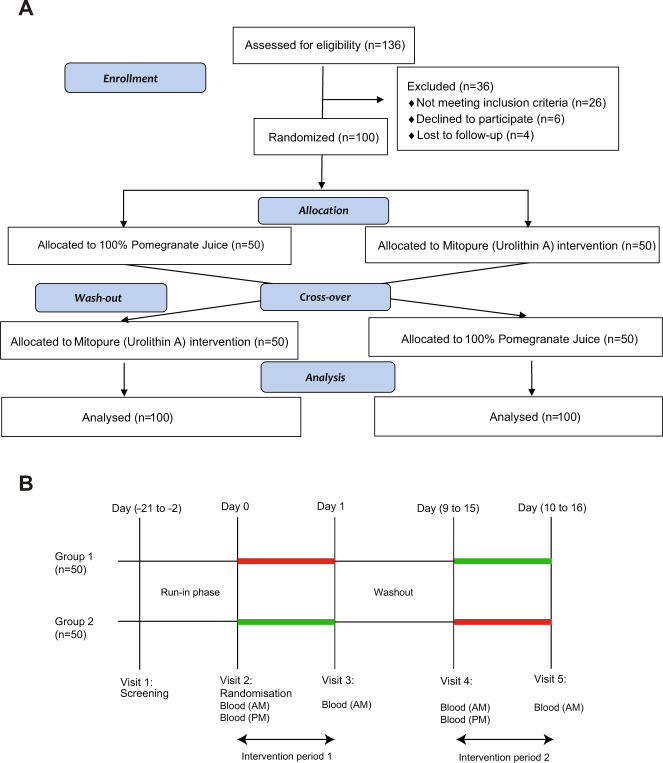
A two-period, crossover, randomized trial study design in healthy adults comparing PJ dietary challenge to Mitopure supplementation. **A** The corresponding CONSORT diagram is represented. A total of 136 subjects were screened in the study, following which randomization occurred (*n* = 100) to one of the two study interventions in sequential manner. Following a washout period of 8–14 days, the crossover period occurred in which subjects took the second intervention for comparison of bioavailability. All subjects completed the study and there were no drop-outs or major protocol violations resulting in all data being analyzed. **B** Simplified schema of the clinical study design. In total there were five study visits: Visit 1 (screening), Visit 2 (randomization, blood collection at baseline (T0) and 6 h following intake (T6)), Visit 3 (blood collection 24 h after intake of 1st intervention (T24)). The three first visits were followed by a washout period of 8–14 days after which the crossover intervention occurred with the corresponding blood draws (Visits 4 and 5).

The trial was a single-center, two-period, crossover, randomized, open label study. It was approved by an independent institutional review board (Advarra IRB, Columbia, MD, USA) to be conducted at a clinical research site (Atlantia Food Trials, Chicago, IL, USA) and is registered as NCT04160312. Volunteers were recruited (from October 2019 to January 2020) through the clinical site database, social media platforms, general practitioners’ offices, and advertisements in local newspapers. From 136 screened participants, 100 successfully screened subjects were randomized on site to either the PJ or Mitopure intervention group (Fig. 1A). All subjects completed the study (last subject completed in February 2020) and there were no drop-outs. Among the randomized study participants, 68 were females and 32 were males (Supplementary Table 1.1). All were healthy and aged between 18 and 80 years, with a majority of either Caucasian, African-American, or Hispanic ethnicity (Supplementary Table 1.2). Subjects were recruited to allow for a minimum of 20% representation in the following age groups: 18–40, 41–60, and 61–80 years. The remaining 40% were distributed among the different age groups. The average age of the population was 48.65 ±14.67 years (mean ± SD; Supplementary Table 1.3) with an average BMI of 29.76 ± 7.72 (mean ± SD; Supplementary Table 1.4). The study involved five visits to the site over a 5-week period with a run-in phase of 3 weeks followed by the first period of intervention. Following this first period, a washout period of 8–14 days was implemented to ensure complete elimination of UA from circulation, as observed previously [6], after which subjects were administered the crossover dietary intervention (Fig. 1B).

### Inclusion and exclusion criteria

To be eligible for inclusion, subjects needed to be in general good health defined as the absence of any chronic ailments and as determined by the study medical doctor, and to have given voluntary, written, informed consent to participate. Subjects also needed to be willing to consume the investigational products, complete screening questionnaires, and complete all clinic visits during the study. Subjects were excluded if they had presence of any chronic medical conditions and clinically significant abnormal laboratory results at screening. Individuals deemed to be cognitively impaired and unable to give informed consent were excluded as well. Women who were pregnant, breastfeeding, or planning to become pregnant during the course of the trial, and subjects who had taken antibiotics within the previous 30 days of the study screening, were also excluded. Subjects were instructed to follow their usual diet and exercise routine and to not consume any other dietary supplements that could interfere with the assessment of the study products for the duration of the study. Subjects completed a food-frequency questionnaire (FFQ) during the screening visit to assess their dietary intake. The FFQ used for this purpose was the DHQ-III [15].

### Investigational products and randomization

Subjects who successfully passed the screening procedures were randomized on a 1:1 basis to receive either Mitopure (500 mg UA) oral supplementation or 8 fl. oz (~240 mL) of PJ. Natural precursors to UA (EA and punicalagins) and UA levels were assessed in each batch of the PJ and Mitopure administered using calibrated and validated HPLC methods. Randomization was carried out by a study-independent statistician using a computer-generated program to create a block randomization list. Each subject was assigned a unique randomization number. Participants were randomized to one of the following sequence groups as follows:

- Group 1: Mitopure – Pomegranate Juice
- Group 2: Pomegranate Juice – Mitopure

The investigation products were the Mitopure powder, a fruit-flavored powder containing 500 mg of pure UA and ingredients that include blueberry, raspberry fruit powder, pomegranate natural flavoring, and fiber and 8 fl. oz (~240 ml) of a commercially available 100% PJ (POM Wonderful, Los Angeles, CA, USA). Intake happened onsite and was supervised by the research staff to ensure 100% compliance. Mitopure powder was admixed into a vanilla-flavored commercial yogurt (4 fl. oz) and the PJ was served chilled. Participants were asked to consume the investigational product within 10 min. The time of the first sip of PJ and spoonful of yogurt intake containing admixed Mitopure was recorded as the time of product ingestion in the study database.

### Blood and fecal samples collection

Plasma and dried blood spot (DBS) samples were collected from all subjects at baseline (T0), at 6 h (T6), and 24 h (T24) after the intake. During the screening visit, approximately 16 mL of blood was collected to assess clinical, laboratory, and hematology parameters. Following that, at each visit and time point, an 8 mL blood sample was drawn into K2-EDTA coated tubes (Supplementary Table 2). The blood samples were gently inverted several times to ensure complete mixing with the anticoagulant. The exact time of sample collection was recorded in the study database. Within 30 min of collection each blood sample was centrifuged at 1500 g for 10 min at 4 °C. Within 30 min of centrifugation the top layer of human plasma was transferred into a prelabelled polypropylene tube containing approximately 1500 μL of plasma. Tubes from each time point were capped immediately and the plasma was frozen at −80 °C for storage. DBS were also collected on blood collection cards (Whatman Filter Paper 903 spots card). To collect the sample, a fingerpick with a lancet was performed and three to four blood spots (each containing approx. 20–40 mL of whole blood) were spotted on each DBS card. The cards were dried and stored in sealable biohazard foil bags containing desiccant at room temperature until analysis. Each participant was also supplied with a stool sample collection kit (OMNIGene OMR-200, DNA Genotek, Kanata, ON, Canada) at their screening visit to collect a sample at home prior to study visit V2. Upon return to the study site, the stool samples were stored at −80 °C.

### UA bioavailability measurements

A total of 600 human plasma samples were analyzed from the study (i.e., six samples per subjects with three time points per intervention: T0, T6 hours, and T24 hours) over different batches. Each batch included eight calibration samples, at least two blank samples, one double blank plasma sample, as well as three different quality control samples in duplicate. The inter-batch precision was between 3.1 and 12.3%, whereas the inter-batch accuracy was in the range from 96.6 to 99.3% of nominal concentration. Measurements were performed according to the FDA Guidance for Industry and EMA guidelines on bioanalytical method validation as previously described [10]. The quantification of UA and its metabolites, i.e., UA glucuronide and UA sulfate in plasma, was performed by column separation with reverse phase chromatography using an Agilent 1200 HPLC system (Agilent Technologies, Waldbronn, Germany) followed by detection with a TSQ Vantage triple-stage quadropole MS/MS (ThermoFisher Scientific, San Jose, CA, USA) in the selected reaction mode. The plasma samples were diluted with 200 µL water containing 0.1% formic acid, and thereafter fortified with internal standard. A solid-phase extraction with a Bond-Elut focus plate (Agilent Technologies, Waldbronn, Germany) using water as washing solvent and MeOH as elution solvent was used for sample cleanup, and after elution the extraction solvent was evaporated. Subsequently, the sample was reconstituted in 200 µL of a water/methanol mixture (1/1, v/v) and 50 µL was injected into the HPLC MS/MS system. Separation was achieved using a C18 reverse phase column (YMC Co, Ltd., Kyoto, Japan). For the measurements of parent UA in plasma, a validated method was developed. Analysis and quantification were performed using a characterized reference sample of synthesized UA, as the reference item, as well as labeled 13C6 UA, as an internal standard. For the preparation of the internal standard solution 13C6 UA was dissolved in acetonitrile/dimethylsulfoxide (1/1, v/v) to a concentration of 1.00 mg/mL and subsequently diluted to 100,000 ng/mL. Further dilution to a concentration of 2500 ng/mL was done in methanol. In case signal interference compromised the quantification of the analyte and/or the internal standard, the sample was repeated (if sufficient sample volume was available). The concentration of the repeated analysis was reported in case of the absence of the interfering signal. Otherwise, no value was reported for that sample. For quality control in general, 10% of the samples were reanalyzed. Repeat samples were chosen randomly by the analyzing laboratory from the samples belonging to the different study interventions. To measure the levels of UA glucuronide in both plasma and in human whole blood collected in DBS validated methods were developed for each of these matrixes. Analysis and quantification of UA glucuronide was performed for both of these matrixes using a synthesized reference sample consisting of and equal mix of the two isoforms of UA glucuronide as the reference item, as well as labeled 13C6 UA glucuronide as an internal standard. The measurement of UA sulfate in plasma was performed using a validated method, using a characterized reference sample of synthesized UA sulfate as the reference item, as well as labeled 13C6 UA sulfate as an internal standard. The limit of quantification in plasma was 5.00 pg/ml for parent UA and 5.00 ng/ml for both the UA glucuronide and UA sulfate its metabolites. An analytical method to determine UA glucuronide in human whole blood collected by DBS using liquid chromatography coupled to mass spectrometry was also developed and validated. The limit of quantification for UA glucuronide in human DBS samples (6-mm spots) range was 5.00–5000 ng/mL for the analysis of human DBS samples. The quantification of UA glucuronide was performed by column separation with reversed phase liquid chromatography followed by detection with triple-stage quadrupole MS/MS in the selected reaction monitoring mode. The concentration of the analytes was calculated using the internal standardization method. The acquisition and processing of data was performed using LCquan version 2.5.6 and Xcalibur version 2.0.7 (Thermo Fisher Scientific, San Jose, CA, USA).

### Quantification of ellagic acid, punicalagins, and UA in commercial grade food products and dietary supplements

Validated HPLC analytical methods were used to detect EA and punicalagin. For determination of the parent UA levels in commercial grade food products and dietary supplements, a validated HPLC analytical method was developed with a limit of quantification of 1.17 ng/ml. The quantification was performed by column separation with reversed phase liquid chromatography followed by detection with a diode array detector.

### Shotgun metagenomic sequencing and analysis of microbiome

DNA was extracted from ~0.1 g aliquots of the fecal samples using the NucleoSpin^®^ 96 Soil kit (Macherey Nagel). A minimum of one negative control was included per batch of samples from the DNA extraction and throughout the laboratory process (including sequencing). A ZymoBIOMICS™ Microbial Community Standard (Zymo Research) was also included in the analysis as a positive (mock) control. Before sequencing, the quality of the DNA samples was evaluated using agarose gel electrophoresis, and the quantity of the DNA was evaluated by Qubit 2.0 fluorometer quantitation. The prepared DNA libraries were evaluated using Qubit 2.0 fluorometer quantitation and Agilent 2100 Bioanalyzer for the fragment size distribution. Quantitative real-time PCR was used to determine the concentration of the final library before sequencing. The library was sequenced using 2 × 150 bp paired-end sequencing on an Illumina platform. A total of 99 fecal samples were sequenced to an average depth of 19.9 M read pairs (Illumina 2 × 150 PE) per sample. On average, 96.5% of the high-quality microbiome reads from a sample were mapped to a reference human gut gene catalog, and on average 200 metagenomic species (MGS) were detected per sample. Shotgun sequencing was successful for all samples, and metagenomic analysis was performed to provide taxonomical and functional abundance profiles. For MGS abundance profiling, a set of 1273 MGS, which have highly coherent abundance and base composition in a set of 1776 independent reference human gut samples, was detected. The analysis is based on the MGS concept [16]. To taxonomically annotate the MGSs, all the catalog genes were blasted to the NCBI RefSeq genome database (October 1, 2018). To annotate at the various taxonomic ranks, different levels of similarity were required (95, 95, 85, 75, 65, 55, 50, and 45% for subspecies, species, genus, family, order, class, phylum, super kingdom, respectively) and a minimum of 80% sequence coverage. The percentage of genes of each MGS that mapped to each species was calculated, and species level taxonomy were assigned to an MGS if >75% of genes could be annotated to a single species. For genus, family, order, class, and phylum, 60, 50, 40, 30, and 25% consistency levels were used, respectively. Furthermore, at species and at genus level, the MGS was not assigned if another set of more than 10% of the genes belonged to a single alternative species/genus. For each MGS, a signature gene set was defined as the 100 genes optimized for accurate abundance profiling of the MGS. However, an MGS was considered detected only if read pairs were mapped to at least three of its 100 signature genes; counts for MGSs that did not satisfy this criterion were set to zero. MGS counts was normalized according to effective gene length (accounting for read length) and then normalized to sum to 100%, resulting in relative abundance estimates of each MGS. Statistical comparisons between groups were performed using the two-sided Mann–Whitney *U* test. To assess differences in the overall species community composition between the study groups (beta diversity), we used permutational multivariate analysis of variance (PERMANOVA) with 1000 permutations. When performing statistical testing on multiple hypotheses, the Benjamini–Hochberg (BH) method was used to control the false discovery rate (FDR) at a level of 10%.

### Safety assessments

During the study, all adverse events (AEs) reported by the subject were documented with respect to onset, severity, resolution, and relatedness to the investigational products by a qualified medical investigator. All AEs occurring during the conduct of the clinical study were recorded in the electronic Case Report Form. All AEs coded via MedDRA and recorded during the study are reported in Supplementary Table 3. Blood pressure, heart rate, and temperature were measured at the screening visit and at all following visits. Safety labs (blood chemistry, hematology, lipid profile, and HbA1c) were measured at the screening visit only.

### Statistical analysis

An independent statistician performed sample size estimates. The study was powered on the primary objective of showing superiority of UA supplementation to PJ consumption to increase UA levels from baseline. With a sample size of 100, the estimated power to detect a 3.5 times higher UA concentration after UA supplementation as compared with PJ consumption was set at 80%. Following the ICH guideline E9, a blind data review meeting was held prior to locking the study database to define the Intent-to-treat (ITT) and Per-Protocol (PP) statistical populations. ITT population was defined as all randomized participants with at least one consumption of either placebo or treatment product. PP population was defined as all randomized participants with complete data for the primary outcome and at least 95% compliance in terms of amount of product consumed with no major protocol deviations as agreed during the blind data review. Participants who dropped out prior to receiving any treatment were excluded. The primary endpoint was the absolute change in UA glucuronide plasma levels from T0 to T24 in the UA group as compared with the PJ group. Primary outcome data were assessed to identify any confounders and carry-over effects from period 1 to period 2 of the trial. If there were no confounders, repeated measures ANOVA was used to detect any difference in UA glucuronide plasma levels between active (Mitopure) and comparator (PJ) over time. Absolute change was measured by comparing UA glucuronide plasma levels at the two time points. For the area under the curve (AUC) analysis, unpaired *t*-test was used to determine if there was a difference in AUC between Mitopure and PJ supplementation for UA glucuronide, UA sulfate, and parent UA. For microbiome sequencing results, statistical comparisons between groups were performed using the two-sided Mann–Whitney *U* test. To assess differences in the overall species community composition between the study groups (beta diversity), PERMANOVA was used. When performing statistical testing on multiple hypotheses, the study used the BH method to control the FDR at a level of 10%.

## Results

### Determination of dietary precursor levels in pomegranate-based supplements and juice

To identify the most suitable interventional product that contained dietary precursors and would lead to the generation of UA, the study measured the levels of UA precursors (EA and punicalagins A and B) in the matrix of several commercial grade pomegranate extract dietary supplements and juice-based products (Supplementary Fig. 1). There was considerable variation, with some products having trace levels and others enriched in either EA or punicalagins. In all, 100% PJ was selected as the comparator as it showed a batch-to-batch consistency of the two-precursor compounds (71 mg punicalagins and 36 mg EA/8 fl. oz). Neither the supplements nor the PJ had detectable levels of UA in their matrix. The Mitopure powder reported a dose of 500 mg of UA within its matrix, with trace levels of precursor compounds from berry fruit powder added as flavoring.

### Compliance to study products and safety

Compliance was high with all participants consuming >95% of the study products. All participants completed the study and there were no drop-outs. No serious AEs were reported during the conduct of the study. There were 26 AE events reported following PJ intake and 15 AEs reported during the period of Mitopure intake (Supplementary Table 3). No particular differences were observed in the prevalence of any AE, most of which were mild and resolved during the study.

### The majority of the study population failed to produce UA following dietary exposure to PJ

**Fig. 2:**
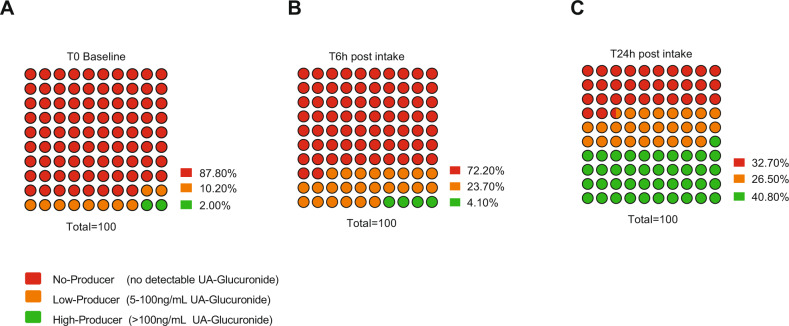
Prevalence of UA producer status in the studied American population. Subjects were categorized into three producer groups based on circulating UA glucuronide levels: non-producers (no detectable levels), low producers (<100 ng/mL UA glucuronide), or high producers (≥100 ng/mL UA glucuronide). **A** At baseline only 12% subjects had detectable levels of UA glucuronide in circulation with only 2% subjects classified as high producers. **B** Six hours following dietary challenge with PJ approx. 28% subjects had detectable levels of UA glucuronide, with only 4% subjects high producers. **C** One day (T24 hours) after the PJ intake, approx. 40% of subjects had become high converters, whereas 60% still converted poorly or failed to convert the dietary precursors to UA.

Only 12% of adults in the studied population had detectable levels of UA glucuronide at baseline, i.e., real-life free-living state (Fig. 2A). Subjects were categorized into three producer groups based on circulating UA glucuronide levels: non-producers (no detectable levels), low producers (<100 ng/mL), or high producers (≥100 ng/mL). Six hours after drinking the PJ, plasma levels showed most subjects still did not display gut-mediated production of UA: 24% of subjects had detectable, but low levels of UA glucuronide in circulation and only 4% subjects were above the 100 ng/mL threshold (Fig. 2B). The conversion of pomegranate precursors into UA was markedly increased 24-h after drinking PJ; however, still only ~40% of subjects displayed UA glucuronide plasma levels >100 ng/mL. The remaining 60% were still either unable to convert (33%) or were poor converters of UA (27%) (Fig. 2C).

### Gut microbiome plays an essential role in defining UA producers

**Fig. 3:**
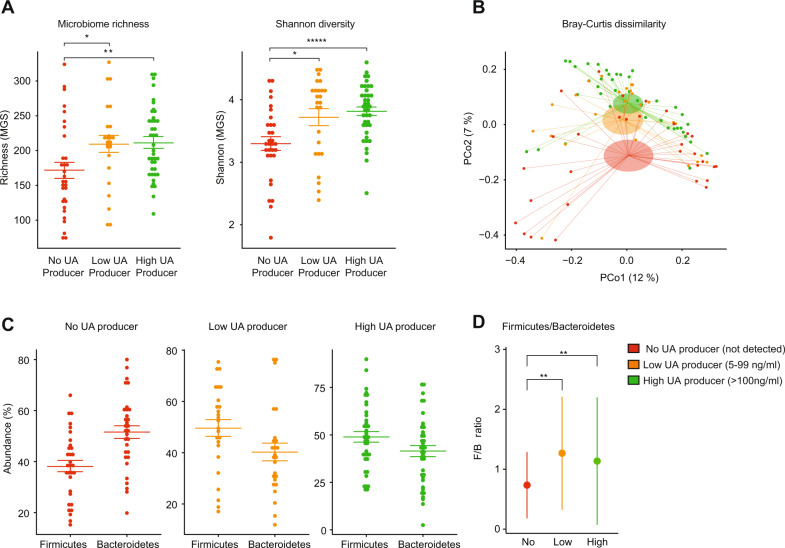
Gut microbiome differences between UA producers and non-producers. **A** Boxplots showing differences in metagenomics species (MGS) for richness (left panel) and Shannon diversity (right panel) between groups with no, low, and high UA producer status. All groups were compared pairwise by Mann–Whitney U test (N = 99). *p ≤ 0.05; **p ≤ 0.01; ***p ≤ 0.001; *****p ≤ 0.00001. **B** Principal coordinate analysis (PCoA) based on Bray–Curtis dissimilarities among samples, calculated based on the MGS abundances. Samples are color coded by the UA producer status. Each sample is connected to its group centroid by a thin segment line. The ellipses cover two standard errors of the mean of the group centroids, i.e., they illustrate the certainty of the group centroid positions. The *x*- and *y*-axis labels indicate the microbial variance explained by the first two principal coordinates**. C** Relative abundance (in %) of phyla Firmicutes and Bacteroidetes that significantly differed in abundance in producer of UA compared with no producer. Boxes represent interquartile range (IQR), with the inside horizontal line representing the median. Whiskers represent values within 1.5× IQR of the first and third quartiles. **D** Firmicutes/Bacteroidetes ratio (F/B ratio) for each group shown as median (IQR). **p ≤ 0.01 (Mann–Whitney *U* test).

Shotgun sequencing determined microbiome composition from the fecal samples of individuals producing UA at different levels following PJ intake. This metagenomic analysis compared the abundance of MGS and genes in subjects from the no, low, and high producer groups. Metagenomic profiles determined the microbiome alpha diversity, which indicates variation of microbes in a single sample. Alpha diversity was assessed both as microbiome richness (number of species or genes observed in a sample) and microbiome variability, and was quantified using the Shannon index [17]. The Shannon index accounts for the number of species or genes in a community, and also their relative abundance. Results showed significantly higher richness in the microbiome Shannon index in both the low producers and high producers, when comparing each with the no producer group, for both MGS (Fig. 3A) and gene-based (Supplementary Fig. 2) measures. A second analysis determined changes in beta diversity, which accounts for differences in relative abundance of MGSs among samples, using Bray–Curtis dissimilarity. Bray–Curtis dissimilarity can range between 0 and 1, where 0 means that the two samples have identical compositions (they share all species at the same relative abundance), and 1 means that the two samples are completely different (they do not share any species). A principal coordinate analysis of the Bray–Curtis dissimilarities (Fig. 3B) showed a shift in the overall microbiome composition when comparing non-producers with low (*p* = 0.048) and high producers (*p* = 0.001), as shown by the clear segregation of the groups. No clear separation and an overlap were observed between the groups of low producer and high producer. In summary, the alpha and beta diversity results indicate that the ability to convert UA from its precursors is significantly associated with a higher microbiome richness and overall composition.

### UA producer and non-producer exhibit differentially abundant gut microbiome taxa

In line with the alpha and beta diversity results, analysis found differentially abundant taxa in UA producers (low and high production status) compared with non-producers. At the phylum level, abundancy of Firmicutes (F) and Bacteroidetes (B) was assessed, as increased F/B ratio is associated with several markers of gut and organismal health [18–20]. Both groups (low and high producers) capable of producing UA showed a higher abundance of Firmicutes with respect to Bacteroidetes, while the opposite was observed for the no producer group (Fig. 3C). The F/B ratio was significantly higher in UA producers compared with non-producers (Fig. 3D). Next, analysis of changes at the levels of species (MGS), and of higher taxa among the three UA producer status groups, found four MGSs and two phyla with significantly different abundance or prevalence between the low producer group and the non-producer group (Supplementary Fig. 3A). Notably, all taxa that were significantly different between the non-producer and low producer group were also significantly different between the non-producer and high UA producer group. High producers showed even larger differences, as 57 MGSs and 33 taxa (14 genera, 15 families, 4 phyla) had significantly different abundance or prevalence in the high producer group compared with the no producer group (Supplementary Fig. 4). In particular, a high abundance of species belonging to the *Clostridiales* and *Ruminococcaceae* family was found in the high UA producer group. Of note was also the increased abundance of *Akkermansia muciniphilia* in the microbial high UA producer group compared to the low and non-producers (Supplementary Fig. 3B).

### Direct oral supplementation with Urolithin A delivers significantly higher levels than PJ

**Fig. 4:**
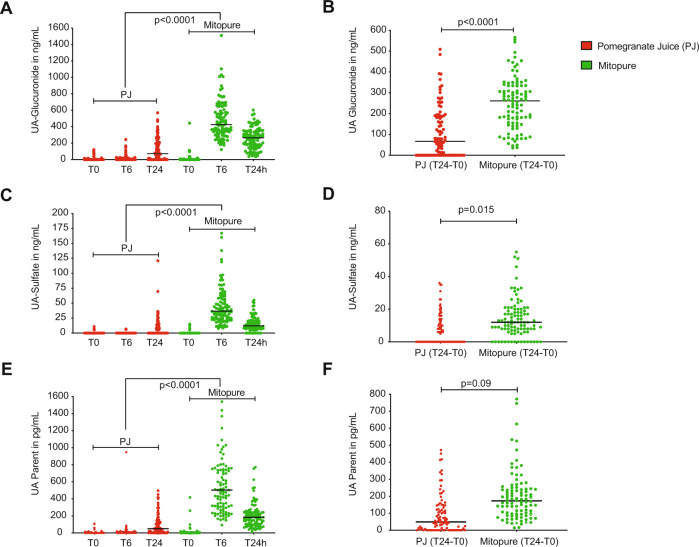
Mitopure supplementation delivers significantly higher plasma UA levels compared to PJ. Pharmacokinetic profiles at T0, T6 (6 h), and T24 (24 h) and mean absolute change in levels from T0 to T24 (primary outcome of study) between the two interventions of PJ and Mitopure supplementation for UA glucuronide (**A**, **B**), UA sulfate (**C**, **D**), and parent UA (**E**, **F**) showing significantly higher plasma levels of UA and its metabolites with Mitopure supplementation compared to PJ dietary challenge (*N* = 100). All data are analyzed using a repeated measure ANOVA (**A**, **C**, **E**) and an unpaired *t*-test (**B**, **D**, **F**).

**Fig. 5:**
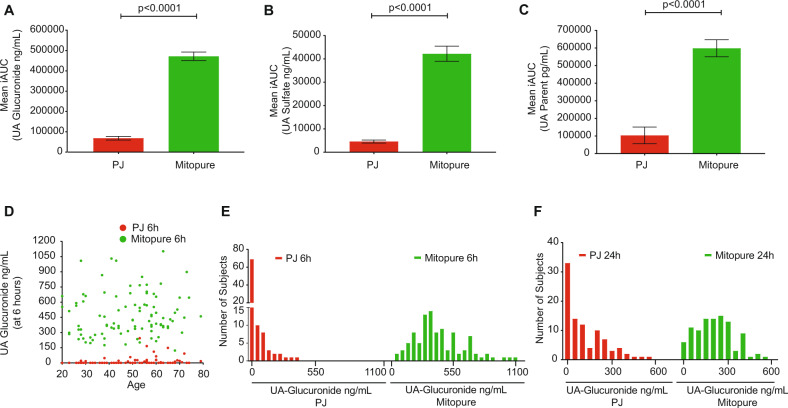
Mitopure supplementation delivers >6-fold higher exposure to UA compared to PJ and achieves consistent levels across the adult population. Mean incremental area under the curve (iAUC) within a day following intake of Mitopure compared to the consumption of a glass equivalent of 100% PJ, for UA glucuronide (**A**), UA sulfate (**B**), and the parent UA (**C**) showing higher exposure to UA and its metabolites with Mitopure compared to PJ (*N* = 100). All data are expressed as mean ± SEM and analyzed using an unpaired *t*-test. Correlation of UA glucuronide levels across different age groups at 6 h following either PJ intake or direct UA supplementation (**D**). Frequency distribution of UA glucuronide levels observed across the population following both PJ and direct UA supplementation at 6 h (**E**) and 24 h post intake (**F**).

In the PJ intervention, the baseline (T0) mean UA glucuronide levels were 5.48 ± 19.97 ng/mL, slightly increasing to 12.84 ± 36.34 ng/mL at the 6 h time point and rising to 110.47 ± 131.6 ng/mL 24 h following PJ intake. In comparison, during Mitopure supplementation, the mean baseline (T0) levels of UA glucuronide were 9.57 ± 47.78 ng/mL; at 6 h UA glucuronide concentration peaked in circulation to 480.75 ± 238.03 ng/mL and then declined to 255.52 ± 129.38 ng/mL 24 h after Mitopure intake (Fig. 4A and Supplementary Table 4). The absolute change in UA glucuronide levels from baseline to T24 (primary outcome of study) between PJ and Mitopure supplementation was investigated to determine plasma levels of UA a day following intake. The analysis showed that circulating levels of UA glucuronide were significantly higher (*p* < 0.0001; 2.4-fold higher mean level) with Mitopure supplementation compared with drinking PJ (Fig. 4B). Comparable plasma profile and absolute change in T24 to baseline levels were seen for UA sulfate (Fig. 4C, D) and parent UA (Fig. 4E, F). The washout period was considered effective and not to have influenced the results during the crossover design, as UA has a half-life of ~24 h with elimination following a single oral dose occurring after between 3 and 4 days [6]. Total exposure (i.e., incremental area under the curve (iAUC)) to UA and UA conjugates was also calculated following the two interventions. The plasma levels of UA glucuronide (mean iAUC; *p* < 0.0001) as assessed by the 6 and 24 h time points (Fig. 5A) were six-fold higher in the Mitopure group than the PJ group. Similar results were observed for UA sulfate (Fig. 5B) and for the parent UA (Fig. 5C). Age had no significant impact on circulating levels of UA following either the PJ or Mitopure intake (Fig. 5D). The 40–60 and 60–80 year age groups did show marginally higher UA levels, as did subjects with normal BMI compared with overweight subjects, but these differences were not statistically significant (Supplementary Fig. 5A, B). Comparing the range of UA glucuronide levels seen across the two interventions over time (Fig. 5E, F), direct UA supplementation delivered uniform circulating UA levels across the population. It took much longer to reach peak circulating levels of UA in participants that were able to perform the natural conversion of dietary precursors to UA and its conjugates than following direct UA supplementation. We also validated the UA glucuronide plasma results with those of DBS samples collected at the same time points. An excellent correlation (Pearson coefficient=0.97) in UA glucuronide concentrations was observed between DBS and plasma values following UA supplementation (Supplementary Fig. 6A, B).

## Discussion

This is the first study to compare the levels of exposure to UA obtained via natural dietary exposure to precursors vs. direct dietary supplementation with UA, and to evaluate the prevalence of natural UA producers across a significant sample size of a hundred healthy adults in a major metropolitan area in USA. This study has led to a further understanding of the complexity of obtaining UA from the diet alone due to dietary differences and the diversity of the microbiome among the general population. Prior to this report, there have been several studies of much smaller scope performed in southern Europe that have explored the identities of the urolithin metabolites produced in individuals following dietary challenges of pomegranate, berries, and nuts rich in ET [1, 3, 13, 21]. These studies have led to the detailed chemical characterization of the family of urolithin metabolites produced following ellagitannin consumption as well as an appreciation of the natural ability to produce these different urolithins, which was observed to range from 30 to 60%. In the studied cohort, only 12% participants had circulating levels of UA at baseline and ~40% significantly converted the dietary precursors to UA after the PJ dietary challenge (Fig. 2).

The current evaluation greatly expands our understanding of human exposure to UA during the initial 24 h after intake of foods containing UA precursor compounds such as PJ and provides an insight on the dosing of natural foods required in order to achieve biologically active levels of UA in the plasma. We have also characterized the gut microbiome of UA producers vs. non-producers and have been able to identify distinct features that determine the ability to produce UA. Gut microbiome make-up determined UA producer status. Non-producers clearly lacked the richness and diversity needed to transform the polyphenolic dietary precursors into UA and had a lower F/B (*Firmicutes* to *Bacteroidetes*) than high producers (Fig. 3). This is particularly relevant as a low F/B ratio has been proposed as a marker of gut dysbiosis associated with obesity (and chronic disorders such as metabolic syndrome [22] and inflammatory bowel disease [23]).

Previous studies have identified certain gut microbiome groups and species to be associated with urolithin production [3, 24]. These investigations have highlighted the role of certain *Clostridium*- and *Gordonibacter*-linked species in the conversion process of EA and ET into urolithin metabolites. Likewise, in our study many differentially abundant taxa were also detected between the high UA producer group compared with the non-producer group. In particular, species belonging to the *Clostridiales* and *Ruminococcaceae* family were found in high abundance in the high UA producer group. Also, of particular interest is the species *Akkermansia muciniphilia*, a gut residing commensal bacteria often associated with health indicators such as lower BMI, low grade inflammation, and improved metabolic health [25–27]. A significantly increased abundance of *Akkermansia muciniphilia* was observed in high UA producers compared to the no producers (Supplementary Fig. 3B). These results are preliminary and further studies are required in well-defined populations to understand the role of this species and the other taxa in determining UA producer status and the gut-mediated UA conversion process.

Other examples of how foods can be metabolized differently based on gut microbiome include the generation of equol and short-chain fatty acids (SCFAs). Equol is a metabolite generated when gut microflora metabolizes dietary isoflavones such as daidzein [28, 29]. Studies have found that 30–40% of the population can naturally generate equol [30, 31]. Similarly, SCFAs are produced upon exposure to dietary fiber and an inability to produce SCFAs has been intricately linked to microbiome dysfunction that eventually leads to chronic disease development such as inflammatory bowel disease and age-related cognitive impairment [32, 33]. Gut metabolites such as UA, equol, and SCFAs are nutrients made available as the end-product of an optimal gut microbial ecosystem. The insight that a majority of people lack the necessary microbiome to make their own UA reveals a gap in our current approaches to achieve a balance in the natural nutrients provided by a healthy diet. This opens the door to dietary supplementation as a strategy to overcome the deficiencies both in the natural microbiome and in dietary consumption patterns that lead to UA heterogeneity in the population.

In a first-in-human study with direct UA supplementation in healthy elderly it was previously reported that UA glucuronide is the dominant circulating metabolite, in addition to parent UA and UA sulfate, and that peak concentrations are achieved at 6 h after intake with the half-life in circulation being approximately a day. In this study, 500 mg of direct UA supplementation was reported to be safe, bioavailable, and have an impact on cellular and mitochondrial health [6], as such this dose was chosen for the current study. PJ containing ET and EA was chosen as the comparator with the most balanced and highest precursor profile and having good batch-to batch consistency in the levels of these precursors (Supplementary Fig. 1). Also, regular consumption of a glass of PJ is the easiest approach to integrate foods into our daily diets for potential exposure to UA. In the present study, we measured UA glucuronide levels at baseline, at the reported peak of 6 h following direct UA supplementation and finally at 24 h after intake. Absolute change in mean concentrations of UA glucuronide, UA sulfate, and parent UA were significantly higher with the direct supplementation (>30-fold at 6 h and >2-fold higher at 24 h; Fig. 4 and Supplementary Table 4). When considering total exposure to UA during the initial 24 h following intake, 500 mg of UA supplementation led to a greater than six-fold (*p* ≤ 0.0001) higher iAUC to UA glucuronide when compared with PJ (Fig. 5A–C and Supplementary Table 4). Age had no impact on UA generation or absorption as well as UA producer status as levels were consistent across the different age groups of young, middle-aged, and elderly (Fig. 5D) both with direct UA supplementation and following PJ intake. Following PJ intake by UA producers, very few subjects exhibited plasma levels in the range observed with direct UA supplementation within the first 6 h and the levels obtained at 24 h (Fig. 5E, F). The UA and its conjugate levels were also much more variable following PJ intake compared to direct UA supplementation that ensured a consistent and a more uniform distribution in the levels within the population.

We also validated a minimally invasive method to determine UA producer status via few drops of dried blood collected via capillary blood (Supplementary Fig. 6) to plasma levels and found an excellent correlation. Such methods would allow for delivery of precise and calibrated dietary supplementation based on existing nutrient levels achieved via dietary exposure.

Based on the findings of the current study, to achieve the equivalent dosing of 500 mg supplementation of UA from dietary exposure via PJ, on average, an individual would need to drink six glasses (approx.1.5 L) of PJ that contained the necessary dietary precursors. The results observed pose an important question in terms of nutritional practices: is an optimal diet sufficient by itself? And how can someone know whether they are likely to harness the appropriate nutrients from the diet via their gut microbiome? Equally important would be to understand better how to harness the various gut microbiome species that would confer the UA producer capacity to an individual, and measure precisely if one was a natural UA producer or not with minimally invasive methods. Advanced nutrition approaches that allow the delivery of a nutritional bioactive such as UA in a calibrated manner will likely play a key role in filling the gap created by the natural heterogeneity of the gut microbiome to deliver health benefits.

## Supplementary information

Supplementary Figures and Tables

## References

[1] Tomas-Barberan FA, Gonzalez-Sarrias A, Garcia-Villalba R, Nunez-Sanchez MA, Selma MV, Garcia-Conesa MT, et al. Urolithins, the rescue of “old” metabolites to understand a “new” concept: metabotypes as a nexus among phenolic metabolism, microbiota dysbiosis, and host health status. Mol Nutr Food Res. 2017;61. 10.1002/mnfr.201500901 27158799

[2] Garcia-Villalba R, Vissenaekens H, Pitart J, Romo-Vaquero M, Espin JC, Grootaert C (2017). Gastrointestinal simulation model TWIN-SHIME shows differences between human urolithin-metabotypes in gut microbiota composition, pomegranate polyphenol metabolism, and transport along the intestinal tract. J Agric Food Chem.

[3] Garcia-Villalba R, Beltran D, Espin JC, Selma MV, Tomas-Barberan FA (2013). Time course production of urolithins from ellagic acid by human gut microbiota. J Agric Food Chem.

[4] Mertens-Talcott SU, Jilma-Stohlawetz P, Rios J, Hingorani L, Derendorf H (2006). Absorption, metabolism, and antioxidant effects of pomegranate (*Punica granatum* l.) polyphenols after ingestion of a standardized extract in healthy human volunteers. J Agric Food Chem.

[5] Seeram NP, Henning SM, Zhang Y, Suchard M, Li Z, Heber D (2006). Pomegranate juice ellagitannin metabolites are present in human plasma and some persist in urine for up to 48 h. J Nutr.

[6] Andreux PA, Blanco-Bose W, Ryu D, Burdet F, Ibberson M, Aebischer P (2019). The mitophagy activator Urolithin A is safe and induces a molecular signature of improved mitochondrial and cellular health in humans. Nat Metab.

[7] Ryu D, Mouchiroud L, Andreux PA, Katsyuba E, Moullan N, Nicolet-Dit-Felix AA (2016). Urolithin A induces mitophagy and prolongs lifespan in *C. elegans* and increases muscle function in rodents. Nat Med.

[8] Fang EF, Hou Y, Palikaras K, Adriaanse BA, Kerr JS, Yang B (2019). Mitophagy inhibits amyloid-beta and tau pathology and reverses cognitive deficits in models of Alzheimer’s disease. Nat Neurosci.

[9] Singh R, Chandrashekharappa S, Bodduluri SR, Baby BV, Hegde B, Kotla NG (2019). Enhancement of the gut barrier integrity by a microbial metabolite through the Nrf2 pathway. Nat Commun.

[10] Toney AM, Fan R, Xian Y, Chaidez V, Ramer-Tait AE, Chung S (2019). a gut metabolite, improves insulin sensitivity through augmentation of mitochondrial function and biogenesis. Obes (Silver Spring)..

[11] Sandhu AK, Miller MG, Thangthaeng N, Scott TM, Shukitt-Hale B, Edirisinghe I (2018). Metabolic fate of strawberry polyphenols after chronic intake in healthy older adults. Food Funct.

[12] Roberts KM, Grainger EM, Thomas-Ahner JM, Hinton A, Gu J, Riedl K (2020). Dose-dependent increases in ellagitannin metabolites as biomarkers of intake in humans consuming standardized black raspberry food products designed for clinical trials. Mol Nutr Food Res.

[13] Gonzalez-Sarrias A, Garcia-Villalba R, Romo-Vaquero M, Alasalvar C, Orem A, Zafrilla P, et al. Clustering according to urolithin metabotype explains the interindividual variability in the improvement of cardiovascular risk biomarkers in overweight-obese individuals consuming pomegranate: a randomized clinical trial. Mol Nutr Food Res. 2017;61. 10.1002/mnfr.201600830 27879044

[14] Selma MV, Gonzalez-Sarrias A, Salas-Salvado J, Andres-Lacueva C, Alasalvar C, Orem A (2018). The gut microbiota metabolism of pomegranate or walnut ellagitannins yields two urolithin-metabotypes that correlate with cardiometabolic risk biomarkers: comparison between normoweight, overweight-obesity and metabolic syndrome. Clin Nutr.

[15] Subar AF, Thompson FE, Kipnis V, Midthune D, Hurwitz P, McNutt S (2001). Comparative validation of the Block, Willett, and National Cancer Institute food frequency questionnaires: the Eating at America’s Table Study. Am J Epidemiol.

[16] Nielsen HB, Almeida M, Juncker AS, Rasmussen S, Li J, Sunagawa S (2014). Identification and assembly of genomes and genetic elements in complex metagenomic samples without using reference genomes. Nat Biotechnol.

[17] Jost L (2007). Partitioning diversity into independent alpha and beta components. Ecology..

[18] Mariat D, Firmesse O, Levenez F, Guimaraes V, Sokol H, Dore J (2009). The Firmicutes/Bacteroidetes ratio of the human microbiota changes with age. BMC Microbiol.

[19] Wills ES, Jonkers DM, Savelkoul PH, Masclee AA, Pierik MJ, Penders J (2014). Fecal microbial composition of ulcerative colitis and Crohn’s disease patients in remission and subsequent exacerbation. PLoS One.

[20] Verdam FJ, Fuentes S, de Jonge C, Zoetendal EG, Erbil R, Greve JW (2013). Human intestinal microbiota composition is associated with local and systemic inflammation in. Obes Obes (Silver Spring)..

[21] Garcia-Mantrana I, Calatayud M, Romo-Vaquero M, Espin JC, Selma MV, Collado MC. Urolithin metabotypes can determine the modulation of gut microbiota in healthy individuals by tracking walnuts consumption over three days. Nutrients. 2019;11:2483. 10.3390/nu11102483 PMC6835957 31623169

[22] Ley RE, Turnbaugh PJ, Klein S, Gordon JI (2006). Microbial ecology: human gut microbes associated with obesity. Nature..

[23] Kostic AD, Xavier RJ, Gevers D (2014). The microbiome in inflammatory bowel disease: current status and the future ahead. Gastroenterology..

[24] Selma MV, Beltran D, Garcia-Villalba R, Espin JC, Tomas-Barberan FA (2014). Description of urolithin production capacity from ellagic acid of two human intestinal Gordonibacter species. Food Funct.

[25] Derrien M, Belzer C, de Vos WM (2017). Akkermansia muciniphila and its role in regulating host functions. Micro Pathog.

[26] Depommier C, Everard A, Druart C, Plovier H, Van Hul M, Vieira-Silva S (2019). Supplementation with *Akkermansia muciniphila* in overweight and obese human volunteers: a proof-of-concept exploratory study. Nat Med.

[27] Cani PD, Bibiloni R, Knauf C, Waget A, Neyrinck AM, Delzenne NM (2008). Changes in gut microbiota control metabolic endotoxemia-induced inflammation in high-fat diet-induced obesity and diabetes in mice. Diabetes..

[28] Atkinson C, Newton KM, Bowles EJ, Yong M, Lampe JW (2008). Demographic, anthropometric, and lifestyle factors and dietary intakes in relation to daidzein-metabolizing phenotypes among premenopausal women in the United States. Am J Clin Nutr.

[29] Bolca S, Possemiers S, Herregat A, Huybrechts I, Heyerick A, De Vriese S (2007). Microbial and dietary factors are associated with the equol producer phenotype in healthy postmenopausal women. J Nutr..

[30] Liu B, Qin L, Liu A, Uchiyama S, Ueno T, Li X (2010). Prevalence of the equol-producer phenotype and its relationship with dietary isoflavone and serum lipids in healthy Chinese adults. J Epidemiol..

[31] Hall MC, O’Brien B, McCormack T (2007). Equol producer status, salivary estradiol profile and urinary excretion of isoflavones in Irish Caucasian women, following ingestion of soymilk. Steroids..

[32] Arpaia N, Rudensky AY (2014). Microbial metabolites control gut inflammatory responses. Proc Natl Acad Sci USA.

[33] Dalile B, Van Oudenhove L, Vervliet B, Verbeke K (2019). The role of short-chain fatty acids in microbiota-gut-brain communication. Nat Rev Gastroenterol Hepatol.

