# Supplementary material for: Direct supplementation with Urolithin A overcomes limitations of dietary exposure and gut microbiome variability in healthy adults to achieve consistent levels across the population
Source: Eur J Clin Nutr. 2021 Jun 11;76(2):297–308. doi: 10.1038/s41430-021-00950-1 (PMC8821002; doi:10.1038/s41430-021-00950-1)
Supplement: Supplementary file 1 — Supplementary Figures and Tables [file 41430_2021_950_MOESM1_ESM.pdf]

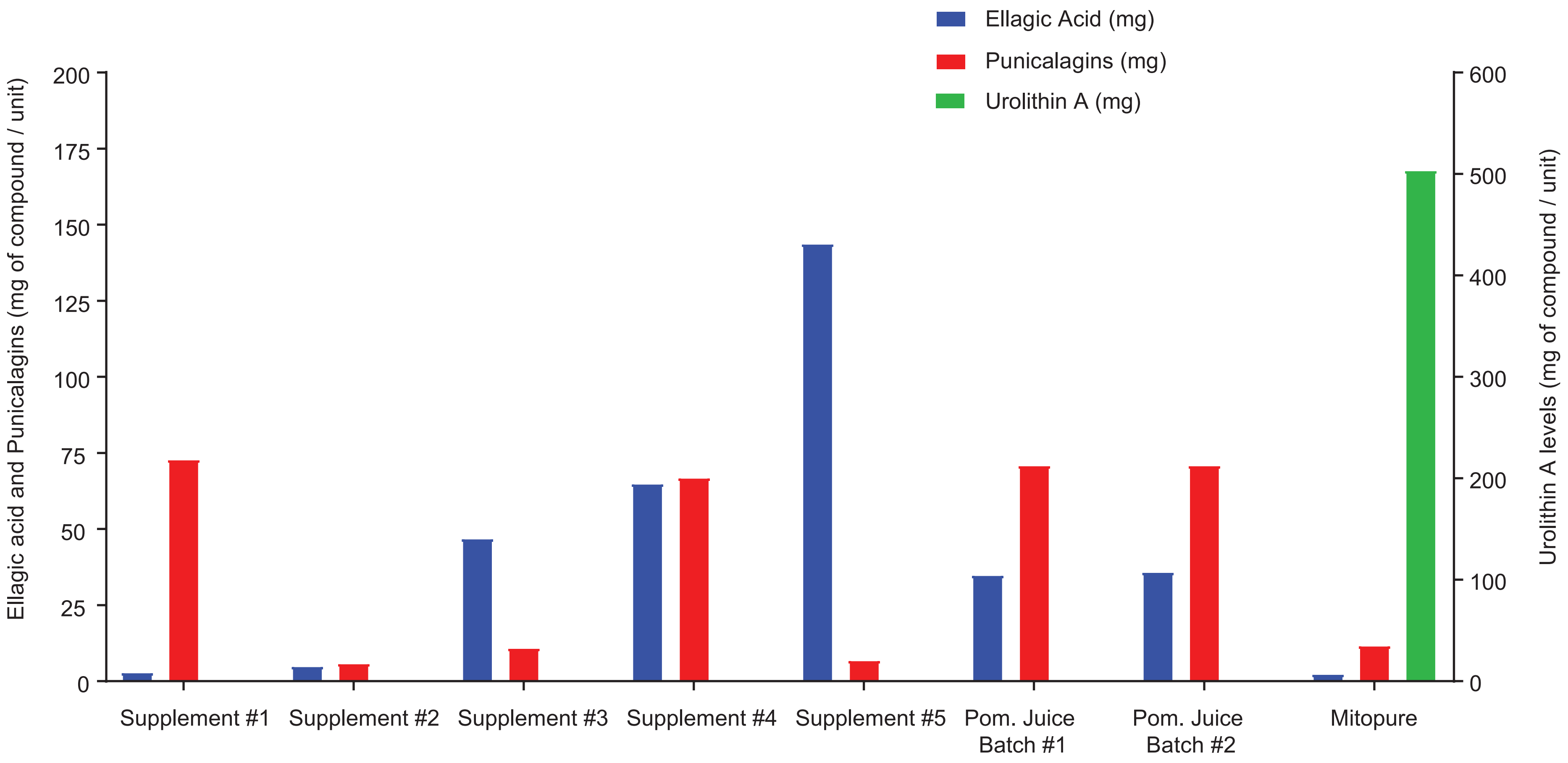

Supplementary Figure 1

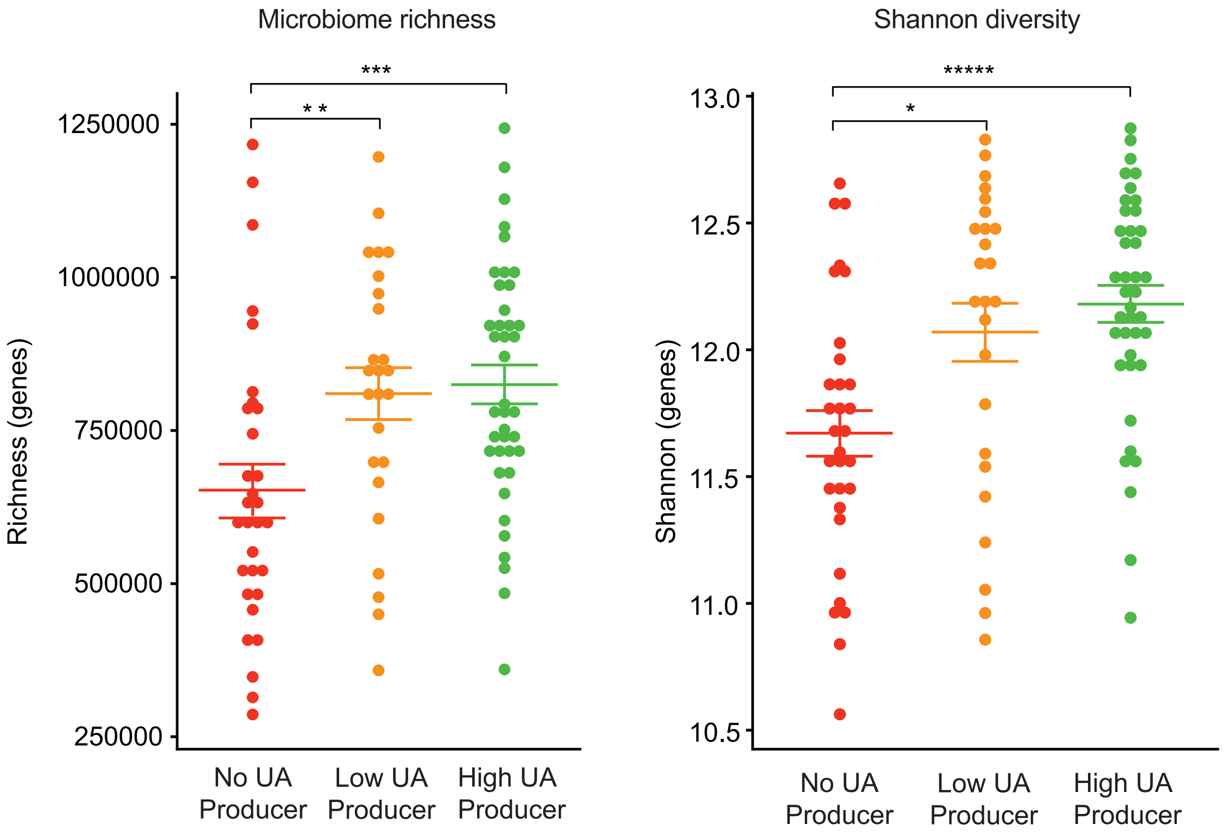

Supplementary Figure 2

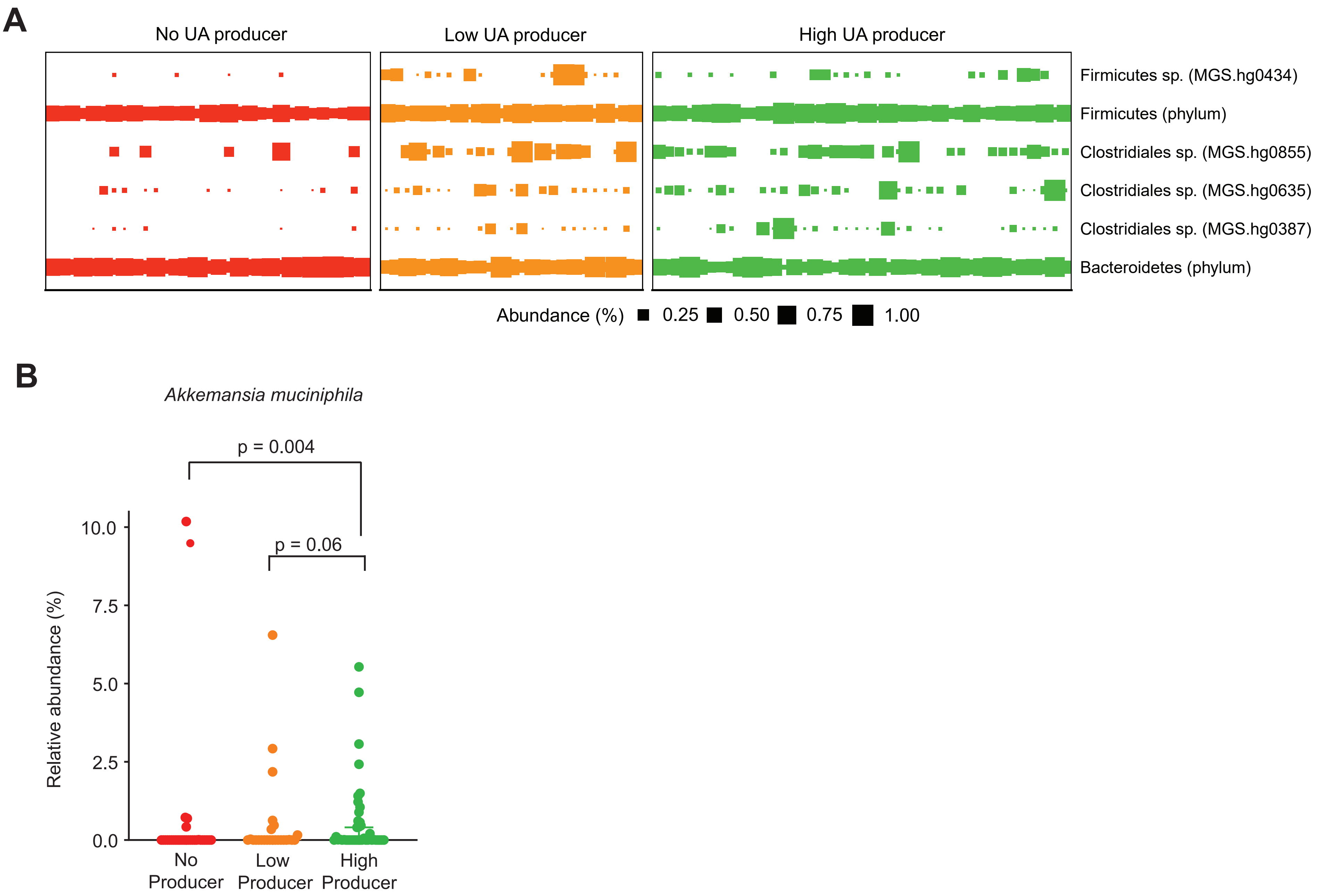

Supplementary Figure 3

A

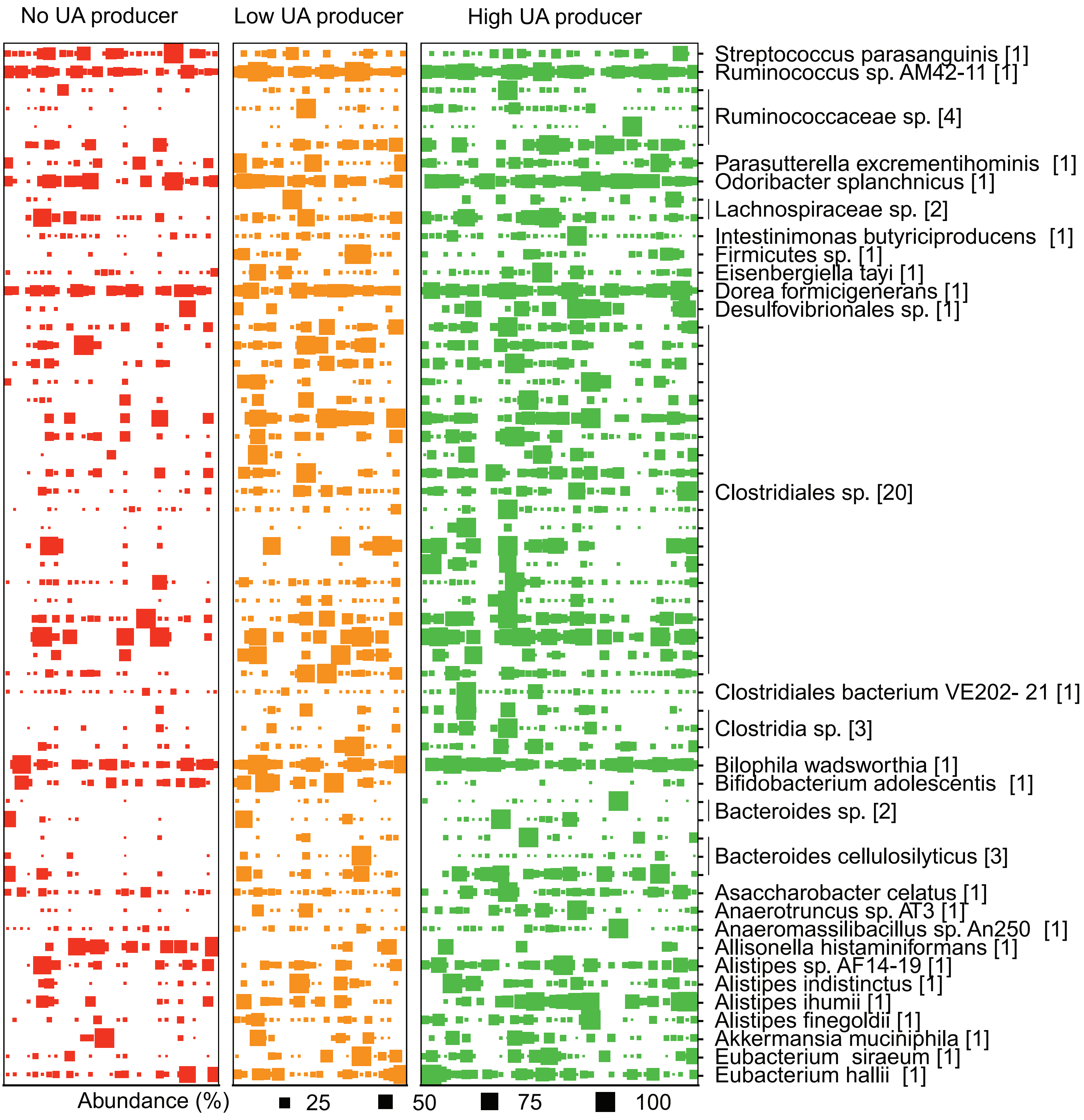

Supplementary Figure 4

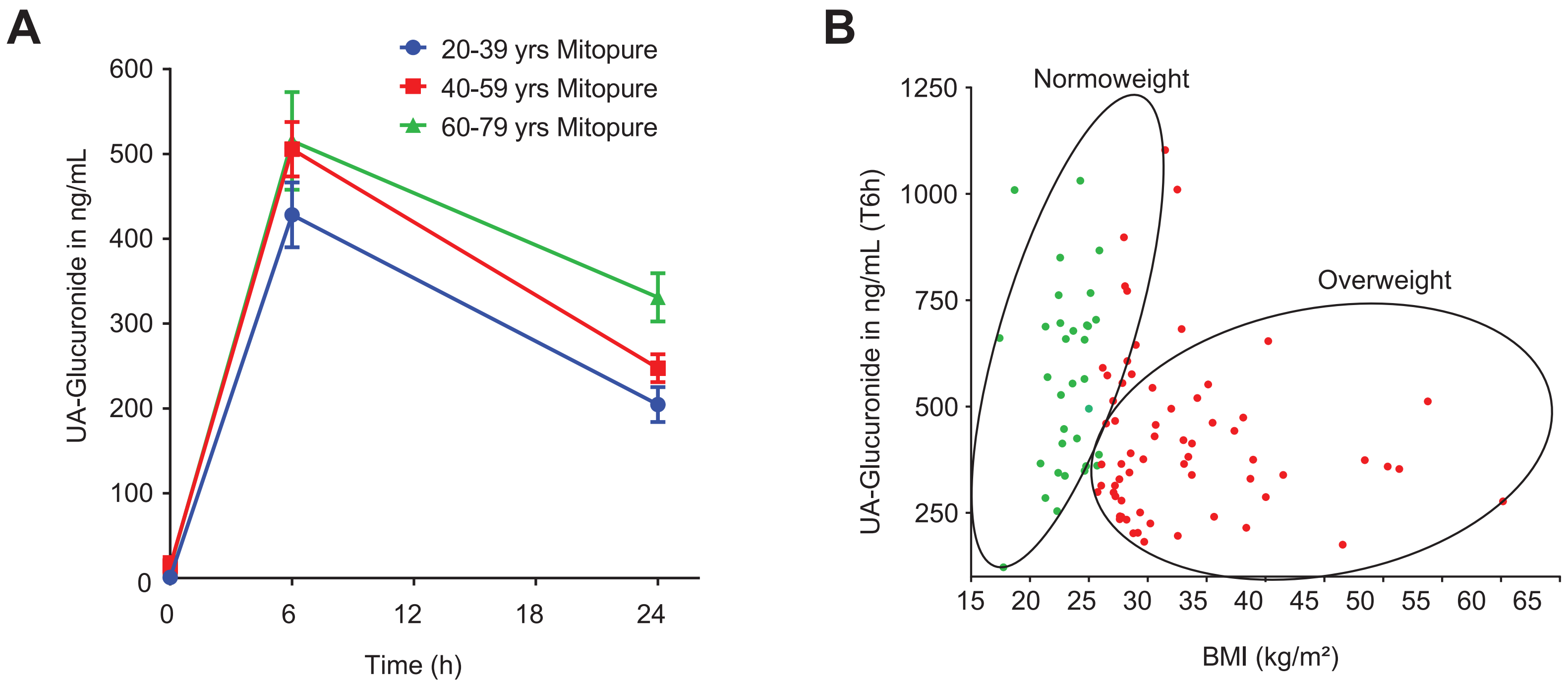

Supplementary Figure 5

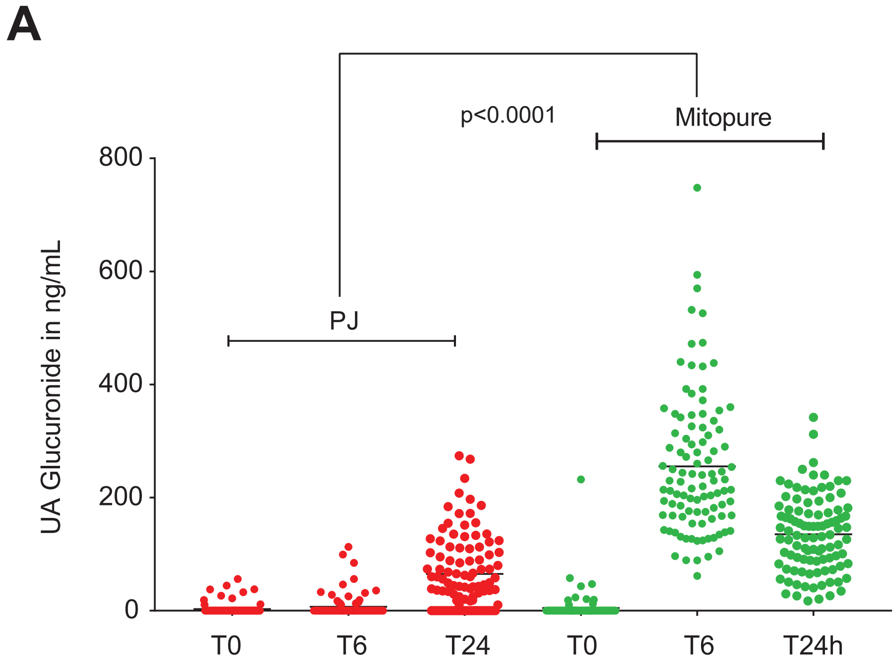

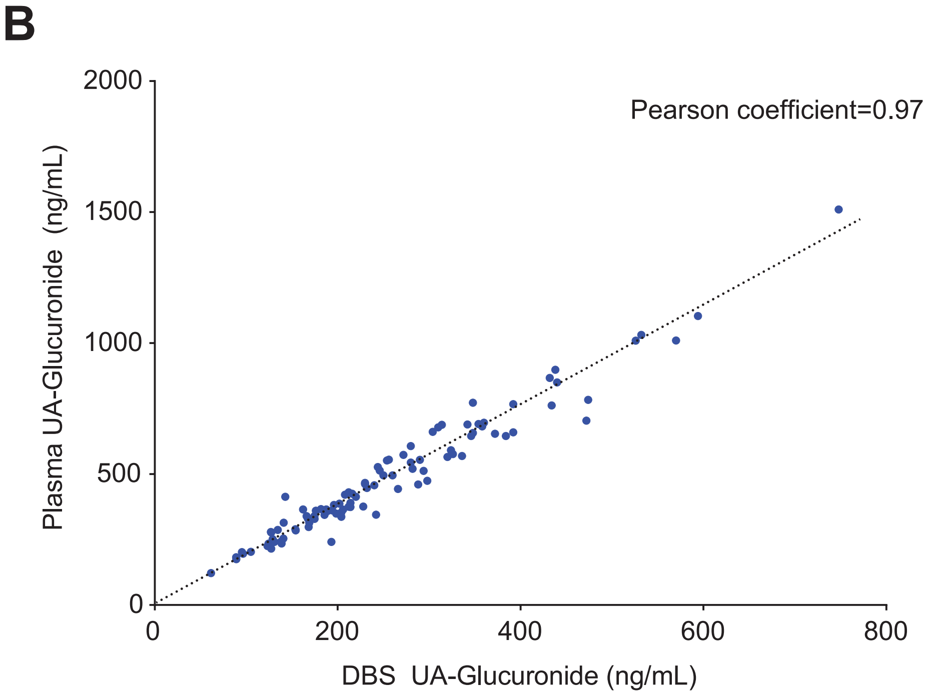

Supplementary Figure 6

Supplementary Figure legends

Supplementary Figure 1

Comparison of ellagic acid, punicalagins (A and B) and UA levels in commercial products. Results are shown as concentration (mg) of precursor present per unit of the product. The levels of the dietary precursors varied across the different dietary supplements that were assessed. Good batch to batch consistency and a balance profile of ellagic acid and punicalagins was observed in the PJ product. UA was only present (at 500 mg concentration) in the Mitopure product.

Supplementary Figure 2

Boxplots showing differences in microbiome genes for richness (left panel) and Shannon diversity (right panel) between groups with no-, low-, and high-UA producer status. All groups were compared pairwise by Mann–Whitney U test (N=99). **P*≤ 0.05; ***P*≤ 0.01; ****P*≤ 0.001, ******P*≤ 0.00001.

Supplementary Figure 3

A) Relative abundance (in per cent) of four MGS and two phyla which significantly differed in abundance or prevalence in UA low-producer compared with no-producer. The relative abundance is shown as the area of each square, scaled to the maximum within each species. **B)** Increased abundance of *Akkermansia muciniphilia* in the microbial high-UA producer group compared to the no-producers group.

Supplementary Figure 4

Relative abundance (in per cent) of MGS which significantly differed in abundance or prevalence in high-producer of UA compared with no-producer. The relative abundance is shown as the area of each square, scaled to the maximum within each species. For each MGS the taxonomy at the best resolution known is shown in parentheses. Number in parenthesis (for e.g., Clostridiales sp. [20]), next to each species label lists the number of different types of species that were found abundantly expressed in UA high-producers

Supplementary Figure 5

A) Plasma levels of UA-glucuronide differentiated by age groups of 20-39 yrs. (blue, n=31); 40-59 yrs. (red, n=42); and 60-79 yrs. (green, n=26) following Mitopure supplementation. No statistically significant differences were observed across the different time-points collected to assess exposure following intake. Data are expressed as mean +/- SEM. **B**) Plasma levels of UA-glucuronide differentiated by body mass index (BMI). Overweight subjects demonstrated lower levels upon Mitopure intake (Not statistically significant) (N = 100). Data analyzed using repeated measure ANOVA.

Supplementary Figure 6

A) Pharmacokinetic profile of UA-glucuronide at T0, T6 and T24 relatively to UA-glucuronide measured with dried blood spots (DBS) (N = 97). Data are expressed as median and analyzed using repeated measure ANOVA. **B)** Comparison of UA-glucuronide levels in plasma and DBS at T6 following Mitopure intake showing an excellent correlation between the two collection methodologies (Pearson coefficient= 0.97).

Supplementary Table 1: Study Population Demographics

Table 1.1. Descriptive frequencies and percentages for sex of sample

		Frequency	Percent	Cumulative Percent
Valid	Male	32	32.0	32.0
	Female	68	68.0	100.0

Table 1.2 Descriptive frequencies and percentages for ethnicity of total population

Ethnicity	Frequency	Percent	Cumulative Percent
African	8	8.0	8.0
African American	22	22.0	30.0
Arabic	1	1.0	31.0
Asian	6	6.0	37.0
Caucasian	49	49.0	86.0
Hispanic	10	10.0	96.0
Indian	1	1.0	97.0
Mixed Ethnicity	3	3.0	100.0

Table 1.3 Descriptive statistics for age profile of sample at Baseline

Parameter	Mean	Std. Deviation	Median	Minimum	Maximum	N
Age	48.65	14.67	51.00	20	79	100

Table 1.4. Descriptive statistics for BMI and weight of total sample and by sex at baseline

Sex		BMI	Weight (kg)
Male	Mean	29.60	90.92
	Std. Deviation	7.57	20.60
	Median	27.37	84.50
	Minimum	21.07	60.90
	Maximum	61.12	166.40
	N	32	32
Female	Mean	29.84	79.74
	Std. Deviation	7.85	21.83
	Median	28.38	74.40
	Minimum	17.63	44.00
	Maximum	54.49	144.10
	N	68	68

Supplementary Table 2: Blood collections during the trial period

Specimen	Blood		
**Test**	**Safety profile**	**Plasma**	**DBS**
Visit 1 - Screening	x		
Visit 2 – T0		x	x
Visit 2 – T6h (+/- 30 mins)		x	x
Visit 3 – T24h (+/- 1 hour)		x	x
Visit 4- T0		x	x
Visit 4- T6h (+/- 30 mins)		x	x
Visit 5 – T24h (+/- 1 hour)		x	x

Supplementary Table 3: Listing of adverse events (AE) by interventions

Product		Frequency	Percent
Pomegranate Juice (PJ)	Anxiousness	1	3.8
	Cold	4	15.4
	Constipation	1	3.8
	Eye irritation	1	3.8
	Finger sprain	1	3.8
	Fractured wrist	1	3.8
	Frequent urination	1	3.8
	Gas	1	3.8
	Headache	3	11.5
	Increased stool frequency	1	3.8
	Migraine	1	3.8
	Muscle pain	1	3.8
	Muscle Pain	1	3.8
	Nasal congestion	1	3.8
	Neck pain	1	3.8
	Osteoarthritis flare up	1	3.8
	Shoulder injury	1	3.8
	Sinus Congestion	1	3.8
	Sore throat	1	3.8
	Stomach pain	1	3.8
	Toothache	1	3.8
	Total	26	100.0
Mitopure	Back pain	1	6.7
	Cold	2	13.3
	Defaecation urgency.	1	6.7
	Feeling lightheaded	1	6.7
	Headache	2	13.3
	Muscle pain	1	6.7
	Myalgia	1	6.7
	Neck pain	1	6.7
	Period cramps	1	6.7
	Root canal procedure	1	6.7
	Sore throat	1	6.7
	Upset stomach	1	6.7
	Urinary tract infection symptoms	1	6.7
	Total	15	100.0

Supplementary Table 4: UA-Glucuronide levels following PJ or Mitopure intake

Descriptive Statistics for UA Glucuronide (ng/mL) at T0, T6hrs and T24hrs and iAUC (over 24 hours)

Product	Time (hours)	Mean	Std. Deviation	iAUC (Mean ± SD)
**Pomegranate Juice (PJ)**	T0	5.48	19.97	**68728** + 91881
	T6	12.84	36.34	
	T24	110.47	131.6	
**Mitopure supplement**	T0	9.57	47.78	**471857*** + 208405
	T6	480.75	238.03	
	T24	255.53	129.38	

*: >6-fold higher exposure to UA observed with Mitopure supplement compared to PJ ($p<0.0001$)
